# Host proteome linked to HPV E7-mediated specific gene hypermethylation in cancer pathways

**DOI:** 10.1186/s13027-020-0271-4

**Published:** 2020-02-03

**Authors:** Nopphamon Na Rangsee, Pattamawadee Yanatatsaneejit, Trairak Pisitkun, Poorichaya Somparn, Pornrutsami Jintaridth, Supachai Topanurak

**Affiliations:** 1 Department of Molecular Tropical Medicine and Genetics, Faculty of Tropical Medicine, Mahidol University, Bangkok, 10400 Thailand; 2 Department of Botany, Faculty of Science, Chulalongkorn University, Bangkok, 10330 Thailand; 3 Center of Excellence in Systems Biology, Faculty of Medicine, Chulalongkorn University, Bangkok, 10330 Thailand; 4 Center of Excellence in Immunology and Immune-mediated Diseases, Department of Microbiology, Faculty of Medicine, Chulalongkorn University, Bangkok, 10330 Thailand; 5 Department of Tropical Nutrition and Food Science, Faculty of Tropical Medicine, Mahidol University, Bangkok, 10400 Thailand

**Keywords:** DNA methylation, Epigenetics, Proteomics, Cervical cancer, Human papilloma virus, Viral oncoproteins

## Abstract

**Background:**

Human papillomavirus (HPV) infection causes around 90% of cervical cancer cases, and cervical cancer is a leading cause of female mortality worldwide. HPV-derived oncoprotein E7 participates in cervical carcinogenesis by inducing aberrant host DNA methylation. However, the targeting specificity of E7 methylation of host genes is not fully understood but is important in the down-regulation of crucial proteins of the hallmark cancer pathways. In this study, we aim to link E7-driven aberrations in the host proteome to corresponding gene promoter hypermethylation events in the hope of providing novel therapeutic targets and biomarkers to indicate the progression of cervical cancer.

**Methods:**

HEK293 cells were transfected with pcDNA3.1-E7 plasmid and empty vector and subjected to mass spectrometry-based proteomic analysis. Down-regulated proteins (where relative abundance was determined significant by paired T-test) relevant to cancer pathways were selected as gene candidates for mRNA transcript abundance measurement by qPCR and expression compared with that in SiHa cells (HPV type 16 positive). Methylation Specific PCR was used to determine promoter hypermethylation in genes downregulated in both SiHa and transfected HEK293 cell lines. The FunRich and STRING databases were used for identification of potential regulatory transcription factors and the proteins interacting with transcription factor gene candidates, respectively.

**Results:**

Approximately 400 proteins totally were identified in proteomics analysis. The transcripts of six genes involved in the host immune response and cell proliferation (*PTMS, C1QBP, BCAP31, CDKN2A, ZMYM6* and *HIST1H1D*) were down-regulated, corresponding to proteomic results. Methylation assays showed four gene promoters (*PTMS, C1QBP, BCAP31* and *CDKN2A*) were hypermethylated with 61, 55.5, 70 and 78% increased methylation, respectively. Those four genes can be regulated by the GA-binding protein alpha chain, specificity protein 1 and ETS-like protein-1 transcription factors, as identified from FunRich database predictions.

**Conclusions:**

HPV E7 altered the HEK293 proteome, particularly with respect to proteins involved in cell proliferation and host immunity. Down-regulation of these proteins appears to be partly mediated via host DNA methylation. E7 possibly complexes with the transcription factors of its targeting genes and DNMT1, allowing methylation of specific target gene promoters.

## Background

Cervical cancer is the fourth leading cause of cancer-related death for women worldwide and a particular problem in developing countries. Over 200,000 people, from approximately 550,000 new cases (50.4%), died in 2017 [1]. Human papillomavirus (HPV) is a causative agent of cervical cancer, and almost 90% of overall cervical cancer cases are linked with high-risk HPV infection [2–5]. About 80–90% of infections are symptomless and removed by the host within a couple of years. However, 10–20% are persistent and can cause carcinoma development [6, 7]. There are more than 100 types of HPV, of which 14 types are cancer-causing and defined as high-risk. From this subset, approximately 70% of all cervical cancer cases are linked to HPV type 16 infection [8, 9].

E6 and E7 are well known HPV oncogenes that induce cervical carcinogenesis [2]. Despite the implementation of screening and vaccination for HPV, HPV-caused cervical cancer remains an issue among patients who have been infected by high-risk HPV. The elucidation of E6/E7 interactions with host proteins and their effect on host physiology could be helpful in defining HPV elimination strategies [10]. The regulation of host physiology by HPV, particularly with respect to E7, has been extensively studied. It has been found that E7 competes with E2F in binding to retinoblastoma protein (pRb), which promotes cell cycle progression and carcinogenesis by increasing degradation of pRb. E7 is also the most prominent HPV protein contributing to host immune dysregulation and evasion [11]. E7 regulates host physiology not only through interaction with host signaling proteins but also by modulating host epigenetics, including DNA methylation. E7 binds host DNA-methyltransferase 1 (DNMT1) and enhances the methylase activity of DNMT1 [12]. DNMT1 is responsible for cytosine methylation in mammals, which downregulates protein expression, playing an important role in gene silencing. E6 also increases the abundance of DNMT1 and p53 contributing to aberrant host gene promoter methylation; another HPV-associated carcinogenesis mechanism [7, 8]. As an oncogenic protein, E7 is likely to methylate a number of genes involved in the hallmark cancer processes, such as cell proliferation and immune evasion. However, the targeting mechanism of E7-directed methylation to certain gene promoters is not fully understood and E7 has no specific binding motif for a host gene promoter. Therefore, we postulated that E7 might bind both to host transcription factors specific to E7 target genes and to DNMT1.

The cell proteome is more closely related to phenotype than readouts from genomics, transcriptomics and epigenetics. Additionally, the relationship between mRNA and protein levels is highly variable between tissue types and cancers and cannot be used for reliable predictions. In this study, we use both proteomics and epigenetics to search for E7-mediated gene methylation, guided by the identification of down-regulated proteins. Our hypothesis; that E7-mediated methylation alters the expression of specific tumor suppressor genes which are important in cervical cancer pathogenesis, is supported by previous studies [13, 14]. One study has mentioned that the hypermethylation of *p16* and *CCNA1* genes stimulate cervical neoplastic progression and contributes to a decrease in cell adhesion molecule 1 (CADM1), which functions in epithelial cell adhesion and is involved in metastasis [14–16]. However, this study did not link this activity to E6 or E7. Numerous studies suggest that DNA methylation occurs at the early stages of cervical cancer and in precancerous lesions [17–19]. HPV persistence alone is insufficient to predict progression of cervical cancer because additional factors participate in tumorigenesis. Therefore, host DNA methylation analysis combined with HPV testing could be a promising option for predicting progression from precancerous to invasive cancer in HPV-positive women [3, 20, 21]. This study was designed to find aberrant E7-mediated DNA methylation events related to cancer pathways to clarify its influence in cervical cancer progression. We hope this study will provide preliminary data for host DNA methylation states in clinical samples, which may identify useful biomarkers.

## Methods

### Plasmid isolation

The pcDNA3.1-E7 (E7) and pcDNA3.1 empty vector (EV) plasmid for mammalian cells expression were kindly provided from Assc. Prof. Pattamawadee Yanatatsaneejit (Human Genetics Research Group, Department of Botany, Faculty of Science, Chulalongkorn University). They were preserved in Luria-Bertani (LB) media (Titan Biotech, India) contained ampicillin antibiotic final concentration 0.1 mg/mL (Merck, Germany) with 40% glycerol (Merck, Germany) and maintained on Luria-Bertani (LB) agar contained ampicillin antibiotic (final concentration 0.1 mg/mL).

### Plasmid transformation

The pcDNA3.1-E7 (E7), pcDNA3.1 empty vector (EV) plasmid and DH5α competent cell were thawed on ice for 5 min. The 100 μL of DH5α competent cell was aliquoted into each 1.5 micro-centrifuge tube. Then 5 μL of E7, EV plasmid were added separately into each tube which containing competent cell followed by gently mixed. Then, tubes were incubated on ice for 5 min. Heat shock method was performed, the tubes were placed in 42 °C thermomixer well (Eppendorf, USA) for 45 s, immediately the tubes were placed on ice for 2 min. Next, 900 μL of SOC medium was added (Biolabs, USA) into each tube and gently incubated the tubes in thermomixer machine for 45 min at 37 °C, 400 rpm. Bacterial cells were collected by spinning down at 8000 rpm for 5 min. The 900 μL of supernatant was discarded and the cell pellet 100 μL was resuspended by pipetting. The 100 μL competent cell contained plasmid was spread on Luria-Bertani (LB) agar (Titan Biotech, India) contained ampicillin antibiotic final concentration 0.1 mg/mL (Merck, Germany). The agar plates were incubated at 37 °C in 5% CO_2_ incubator.

### Plasmid extraction and purification

The positive colonies on Luria-Bertani (LB) agar (Titan Biotech, India) contained ampicillin antibiotic final concentration 0.1 mg/mL (Merck, Germany) were selected and continued cultured in 10 mL Luria-Bertani (LB) broth with 10 μL ampicillin (final concentration 0.1 mg/mL) at 37 °C, 250 rpm shaking incubator overnight. Then, 1000 mL of LB broth with 1 mL of ampicillin (final concentration 0.1 mg/mL) was prepared, 5 mL of overnight cultured bacteria was added then continued cultured at 37 °C, 250 rpm shaking incubator overnight. Plasmid extraction (E7, EV) was done afterward using Maxi Plasmid Kit Endotoxin Free according to the manufacturer’s instructions (Geneaid, Taiwan).

### E7 plasmid detection by PCR and DNA sequencing

The extracted pcDNA3.1-E7 (E7), pcDNA3.1 empty vector (EV) plasmid were determined the concentration by Nanodrop 2000 spectrophotometer (Thermo Fisher Scientific, USA) and investigated E7-inserted gene by PCR using MyTaq™ HS DNA Polymerase kit according to the manufacturer’s instructions (Bioline, UK). The primers are as described. E7 gene forward primer: 5′ GGGCAATTAAATGACAGCTCAG-3′ Tm 54.3 °C and E7 gene reverse primer:5′-GTGTGCTTTGTACGCACAACC-3′ Tm 57.3 °C were used for PCR. The PCR product were observed on 1% agarose (Bio Basic, Canada) by gel electrophoresis to check visual E7 band using GelDoc™ XR+ (Biorad, USA). The extracted pcDNA3.1-E7 (E7) plasmid were sent to Bioneer sequencing service (Bioneer, Korea) for DNA sequencing analysis. The E7 plasmid sequencing results were collected and performed sequence alignment with HPV-16 E7 gene reference sequence (NC_001526.4) from Pubmed database using multiple sequence alignment Clustal Omega website (https://www.ebi.ac.uk/Tools/msa/clustalo/).

### Cell culture medium preparation

The cell culture medium was performed. The preparation of 1 l of MEM supplemented with 10% FBS required 9.5 g MEM (GE Healthcare, USA), 2.2 g NaHCO_3_ (Merck, Germany), 100 mL fetal bovine serum (Gibco, New Zealand) and 900 mL deionized water. The MEM was gently mixed in 1000 mL beaker and steriled filtered through 0.22 μm filter upper cups (Jet Biofil, China) into 1000 mL duran bottle (Duran, Germany). The culture medium was kept at 4 °C, and warmed at 37 °C before use. The preparation of 1 l of DMEM supplemented with 10% FBS required 13.4 g DMEM (GE Healthcare, USA), 3.7 g NaHCO_3_ (Merck, Germany), 100 mL fetal bovine serum (Gibco, New Zealand) and 900 mL deionized water. The DMEM was gently mixed in 1000 mL beaker and steriled filtered through 0.22 μm filter upper cups (Jet Biofil, China) into 1000 mL duran bottle (Duran, Germany). The culture medium was kept at 4 °C, and warmed at 37 °C before use.

### Mammalian cell transfection

The human cervical carcinoma cell line SiHa (HPV type 16-positive), used as E7 expression positive control and C33A (HPV type 16-negative) cell line, used as negative control, were grown and maintained in DMEM supplemented with 10% FBS at 37 °C in an atmosphere of 5% CO_2_, HEK293 (Human Embryonic Kidney 293) cell was grown and maintained in MEM supplemented with 10% FBS at 37 °C in an atmosphere of 5% CO2. In each 6-well plates, SiHa, C33A and HEK293 cells were seeded separately at 2.5× 10^5^ cells/mL in 2 mL of growth medium 24 h before transfection. Then 4 μg of pcDNA3.1-E7 (E7), pcDNA3.1 empty vector (EV) plasmid were diluted in 400 μL of Opti-MEM™ serum-free growth medium (Gibco, New Zealand). TurboFect™ transfection reagent (Invitrogen, USA) was briefly vortexed then the reagent 6 μL was added to the diluted DNA. The mixture was mixed immediately by pipetting followed by incubating 20 min at room temperature. The mixture was evenly distributed at the bottom of the well of 6-well plates containing adherent cells. The plates were gently rocked and incubated at 37 °C in a CO2 incubator. Protein expression analysis was done after 48 h.

### Cell lysis buffer preparation

The cell lysis buffer solution (4% SDS, 0.1 M DTT) were prepared by adding 0.4 g of high-purity SDS (Merck, Germany) and 0.0154 g of DTT (Merck, Germany) to 10 mL of 0.1 M Tris–HCl (Biorad, USA) stock solution at pH 7.6.

### Protein extraction

The transfected HEK293 cell, SiHa and C33A cell line were harvested and washed 3 times with sterile 1X phosphate-buffered saline (1XPBS), by diluting 10X PBS (Merck, Germany) ratio 1:9 to deionized water. The cells were centrifuged at 1000×g for 5 min and removed the supernatant. The cell pellet was snap freezing in a microcentrifuge tube using liquid nitrogen (take extreme caution when doing this). The cell pellet was stored at − 80 °C until cell lysis was performed. The transfected cells and control cell corresponding to 2 × 10^6^ cells (approximately 1 mg pellet of cells) were lysed with 1 mL of lysis buffer. The cell pellet was suspended in the lysis buffer and mixed the solution well using a pipette. Further, the cells were disrupted using a sonicating probe with 20 amplitudes, 10 s pulse on, 10 s pulse off, total 1 min while maintaining the cell lysate at 4 °C and centrifuged at 16,000 x g for 20 min at 4 °C to collect the supernatant. The lysate was heated for 20 min at 56 °C using a heating block to denature the protein. The protein concentration was determined using a BCA assay kit (Thermo Fisher Scientific, USA) as manufacturer’s instructions. The extracted proteins have been kept in − 80 °C for further experiments.

### SDS-page

The protein samples from transfected cells and control were run on SDS-PAGE. The 15% acrylamide separating gel was prepared by adding 2.8 mL deionized water, 3 mL 40% acrylamide (Biorad, USA), 2 mL 1.5 M Tris pH 8.8 (Biorad, USA), 0.08 mL 10% SDS (Merck, Germany), 0.08 mL 10% APS (Sigma-Aldrich, Germany) and 0.008 mL TEMED (Merck, Germany). Then 4% stacking gel was prepared by adding 3.1 mL deionized water, 0.5 mL 40% acrylamide (Biorad, USA), 1.25 mL 0.5 M Tris pH 6.8 (Biorad, USA), 0.05 mL 10% SDS (Merck, Germany), 0.05 mL 10% APS (Sigma-Aldrich, Germany) and 0.005 mL TEMED (Merck, Germany). Finally, SDS-PAGE was started running at 100 V, 1.30 h.

### Western blot analysis

The protein samples in 15% acrylamide gel were transferred to PVDF membrane (Merck, Germany) by western blotting using electricity at 100 V, 2.30 h. The transferred PVDF membrane was later blocked using 5% (w/v) skim milk (HiMedia, India), solved in 1X TBST, 10X TBS pH 7.6 (Biorad, USA) and 0.1% (v/v) tween-20 (Merck, Germany) for 1 h at room temperature. The membrane was probed with primary Ms. Anti-HPV16-E7 mAB (1:200) (Invitrogen, USA) overnight followed by washing for 20 min, 3 times with 1X PBST. The membrane was added with goat anti-mouse IgG, HRP conjugated (1:9000) (Invitrogen, USA) followed by washing for 20 min, 3 times. The Luminata™ Forte Western Blot chemiluminescence HRP substrate (Merck, Germany) was appropriately applied to develop the visual bands.

### 2D − Clean up

The non-specific reagent and detergent were certainly removed from protein samples before sample preparation by using 2D − Clean up kit (GE Healthcare, USA) according to manufacturer’s instructions.

### Protein samples preparation for mass spectrometry

The preparation of 100 mM ammonium bicarbonate buffer was done as follow, 0.158 g ABC (Sigma-Aldrich, Germany) dissolved in 20 mL HPLC grade water (Merck, Germany) then 50 mM ammonium bicarbonate buffer was prepared by diluting 5 mL 100 mM ammonium bicarbonate buffer with 5 mL HPLC grade water. The preparation of 100 mM DTT was done as follow, 0.0154 g DTT (Sigma-Aldrich, Germany) in 1 mL 50 mM ammonium bicarbonate buffer. The preparation of 100 mM IAA was done as follow, 0.0185 g IAA (Sigma-Aldrich, Germany) in 1 mL 50 mM ammonium bicarbonate. The preparation of trypsin from porcine pancreas (Sigma-Aldrich, Germany) digestion enzyme was done by adding 200 μL of 50 mM ABC into 20 μg of trypsin to generate the concentration of 0.1 μg/μL. An aliquot of 100 μg of protein was taken and 50 mM ammonium bicarbonate was added to the protein to fill the total volume up to100 μL. Next, 10 μL of 100 mM DTT (final concentration 10 mM DTT) was added in the tube for 40 min at 56 °C. Protein Alkylation was done by adding 2 μL of 100 mM IAA (final concentration 20 mM IAA) for 60 min in the dark at room temperature. Protein samples were digested later by adding trypsin (ratio trypsin: sample = 1:50) then mixture was incubated at 37 °C overnight. The samples were continued proceeded desalting procedure.

### Desalting by oasis column

The preparation of 50% acetonitrile was done by diluting 5 mL of 100% acetonitrile (Merck, Germany) stock solution with 5 mL of HPLC grade water then 0.1% TFA was prepared by diluting 10% TFA stock solution (Merck, Germany) with 49.5 mL of HPLC grade water (Merck, Germany). Finally, elution buffer, 75% ACN / 0.1% TFA was prepared by adding 7.5 mL of 100% acetonitrile stock solution, 0.1 mL of 10% TFA stock solution and 2.4 mL of HPLC grade water. Oasis columns were placed in 15 mL tube, 1 mL of 50% ACN was added and let it flow, 1 mL of 0.1% TFA was added to equilibrate column, let it flow. The equilibration step was repeated 3 times. The protein samples were added into column and slowly let it flow by gravity. Then 1 mL of 0.1% TFA was added and let it flow. Then, oasis columns were taken and placed in the new clean 15 mL tubes, peptide was eluted by adding 1 mL elution buffer, 75% ACN / 0.1% TFA, let it flow by gravity. The samples were taken to evaporate at speed vacumm centrifuge cincentrator machine (Meditop, Thailand), then kept samples in − 80 °C until further analysis.

### Mass spectrometry analysis

The peptides were identified using an UltiMate® 3000 RSLCnano system (Thermo Fisher Scientific, USA) coupled with Q Exactive Hybrid Quadrupole-Orbitrap mass spectrometer (Thermo Fisher Scientific, USA) through EASY-Spray nano-electrospray ion source (Thermo Fisher Scientific, USA). The samples were loaded with 5–7% Acetonitrile (ACN) in 5 min, 7–45% in 60 min, 45–50% in 5 min, and 50–97% in 5 min, followed by washing at 100% at 300 nL/min flow rate for 90 min. Full MS scan was carried with mass ranges of m/z 200–2000. Precursor ions with + 1 and greater than + 8 charge state were excluded. Fragmentation of precursor ions was performed using Higher-energy collisional dissociation and data acquisition was performed by Thermo Xcalibur 2.2 (Thermo Fisher Scientific, USA).

### Protein identification and peptide quantification

The MS Data were analyzed by Proteome discover version 2.2 (Thermo Fisher Scientific). Spectra were matched with protein database using *Homo sapiens* (Proteome ID: UP000005640) database from Uniprot. Parameters were set to optimized quantification method of label free labeling including; processing workflow were input data of enzyme cleavage as trypsin; Max. Missed cleavage sites were 2; Fragment tolerance and Precursor tolerance were 0.02 Da and 10 ppm, respectively; Modification: Oxidation as dynamic modification and static modification included carbamidomethyl. Consensus workflow was set to result filter for high confidence peptides which PSM was filtered as SEQUEST: XCorr. Chromatographic Alignments included 10 ppm mass tolerance and 10 min was maximum RT shift. The protein results were cut off by false discovery rate lower than 0.5%. Protein abundance was calculated by PD program between E7 transfected and control. In addition, the MS data were proceeding to protein identification and label free quantitation (LFQ) by Maxquant 16.5. In addition, the MS data were proceeding to protein identification and label free quantitation (LFQ) by Maxquant 16.5. For Maxquant database search setting, the criteria were setup as following; peptide tolerance: 20 ppm, MS/MS tolerance: 0.5 Da, Isotope tolerance: 0.1 Da and FDR: 0.05.

### Gene ontology

Protein classification was carried out by their biological processes and molecular functions by Proteome discover version 2.2 (Thermo Fisher Scientific, USA) and FunRich software version 3.1.3.

### Hierarchical clustering of proteins and bioinformatic analysis

The Hierarchical clustering heatmap for identified proteins by Maxquant was generated by Perseus software version 1.6.1.1 (http://www.perseus-framework.org). The matrix generation contained data from protein identification and quantification which analyzed with Maxquant and Proteome Discoverer 2.2 (Demo version). LFQ intensity were transformed to log2 and missing values were imputed with normal distribution (width:3 and down: 1.8) then transformed values were further clustered and performed density estimation for enrichment expression ratio. Scatterplot was also built by Perseus with use transformed LFQ intensity of EV against E7. Thirteen of down-regulated proteins, were found from Maxquant and PD were selected for further measure of mRNA expression and DNA methylation based on gene ontology related cancer. Furthermore, protein annotation was performed with FunRich software version 3.1.3. Custom databases were generated by upload *Homo sapiens* accession downloaded from Uniprot proteome (ID: UP000005640) and protein annotation database downloaded through Perseus annotation (annotations.perseus-framework.org). Dataset of protein down-regulation in each condition was uploaded to compared gene enrichment analysis. Heatmap was generated based on fold enrichment of biological pathways. Putative regulatory transcription factors of DNA methylation genes were predicted and generated heatmap by FunRich database.

### RNA extraction

The plasmid transfected HEK293, SiHa and C33A cell line were performed conventional RNA extraction. Cell culture media was removed from T25 flask and washed with 1XPBS. Then, 1 mL of trizol reagent (Thermo Fisher Scientific, USA) was added to adherent cell and all cells were scraped. Cell lysate was passed up and down through pipette then collected to1.5 micro-centrifuge tube incubated for 10 min then 0.2 mL of chloroform (Thermo Fisher Scientific, USA) was added to 1 mL trizol, the sample was vortexed for 15 s then incubated at room temperature for 3 min. The sample was centrifuged at 12,000 x g 15 min at 4 °C. The upper aqueous phase was collected into the next 1.5 micro-centrifuge tube. RNA precipitation was done by adding 250 μL isopropanol (Merck, Germany) then the RNA briefly mixed and incubated at room temperature for 20 min. The sample was centrifuged at 12,000 x g 10 min at 4 °C. The supernatant was completely removed and the RNA pellet was washed with 1 mL 75% ethanol (Merck, Germany), mixed by vortex. The mixture was centrifuged at 7500 x g 5 min at 4 °C. The washing step was repeated one more time then the supernatant was discarded. The RNA pellet was air dried for 20 min then The RNA pellet was dissolved with 15 μL DEPC-treated water (Thermo Fisher Scientific, USA). The RNA concentration measurement was done by using Nanodrop 2000 spectrophotometer (Thermo Fisher Scientific, USA).

### RT-qPCR primer design

The target genes were selected and further investigated gene expression analysis in RNA level. Oligonucleotides primers for RT-qPCR were designed manually and sent to the company to be further synthesized by Integrated Device Technology (IDT), Inc., USA (Table 1).

**Table 1:** Oligonucleotide sequences and conditions for PCR analyses

Oligonucleotides	Sequence (5′–3′)	Amplicon (bp)	Tm (°C)
*C1QBP*	FW: ATTAGTGCGGAAAGTTGCCGGGG	122	59
	RW: GCTCCTGTTCTTCAACCTTCTGCC		
*CDKN2A*	FW: GAATAGTTACGGTCGGAGGCCG	165	59
	RW: ACCACCAGCGTGTCCAGGAAGC		60
*H2AFY*	FW: GTGAAGTCAGTAAGGCAGCCAGC	137	59
	RW: GTCGATCGAGGCAATGTCAGCC		
*EIF3K*	FW: GGGTATCGACAGGTACAATCCTGAG	119	59
	RW: ACTGGTACAGCTTCAGGACAGCC		
*HIST1H1D*	FW: CTCTGGCCGCGCTTAAGAAAGC	108	59
	RW: CCAGAGTACCTTTGCTCACCAAGC		
*TOMM22*	FW: GCAGCGGCAGATACTTCTAGGAC	117	59
	RW: CCCACTGAGACAGCTCAAACAGC		
*NPM3*	FW: TGACCATCAGGAGATCGCAGTCC	101	59
	RW: GGAAGGTTACAGGTGGTTGGAGC		
*RACK1*	FW: GCGGGGTCACTCCCACTTTGTTA	153	59
	RW: CTCAGCACATCCTTGGTATGGCC		
*TAPBPL*	FW: GGACTGTGGCTTCTCCATGGCA	151	59
	RW: CAGTTGTGCAGGCTCCAGGGTA		
*ZMYM6*	FW: CAGGGGTTGATAAGCCATTCTGTAG	95	58
	RW: GCTGCACATCTTACAGTAGTTTCCC		
*BCAP31*	FW: GTCATCCTTGTGCTGTTGGTCATCG	170	59
	RW: GACAGCAGCAAGGAAAAGCCAGC		
*BTNL8*	FW: GGCCTGTTTGATGTGGAGATCTCTC	98	59
	RW: GATTCCACCTCTCGGCTCAGATG		
*PTMS*	FW: GCTCCAGTCCCATTGGTGGT	213	56
	RW: CTTCGGCAGCTCTCTTCAGC		
*GAPDH*	FW: GCTGAGTACGTCGTGGAGTC	246	56
	RW: TCCTTCCACGATACCAAAGTTGTC		

### Quantitative reverse transcriptase polymerase chain reaction

The target genes were investigated for gene expression by one-step RT-qPCR technique using KAPA SYBR® FAST One-Step RT-qPCR Master Mix (2X) Kit (Kapabiosystems, USA) according to manufacturer’s instructions for Bio-Rad iCycler™ real-time machine (Biorad, USA). The total volume of PCR reaction was 20 μL; KAPA SYBR FAST qPCR Master Mix (2X) 10 μL, 10 mM dUTP 0.4 μL, 10 μM forward primer 0.4 μL, 10 μM reverse primer 0.4 μL, 50X KAPA RT Mix 0.4 μL, Template DNA as required and PCR-grade water (Bioline, UK) up to 20 μL. The qPCR parameters were adjusted properly according to the manufacturer’s protocol (reverse transcription 42 °C for 5 min, enzyme activation 95 °C for 3 min, denaturation 95 °C for 3 s and annealing/extension/data acquisition 60 °C for 20 s for total 40 cycles, dissociation step is depended on instrument guidelines). The melting curves were carried out in each RT-qPCR to verify single-product amplification. The relative level of gene expression was calculated with the Livak method (2 ^−(ΔΔCt)^). The genes protein Glyceraldehyde-3-phosphate dehydrogenase (GAPDH) was used as the reference genes. The measurements were recorded from three technical and three biological replicates for each experimental condition.

### DNA extraction

The plasmid transfected HEK293, SiHa and C33A cell line, DNA isolation from cultured cells were performed by using ISOLATE II Genomic DNA Kit (Bioline, UK) followed the manufacturer’s instructions. The 10^7^ cells were resuspended up in 200 μL lysis buffer GL, 25 μL proteinase K solution and 200 μL of lysis buffer G3 were added then incubated at 70 °C for 15 min. DNA binding condition was adjusted by vortex. Then, the 210 μL of 100% ethanol was added to samples and vigorously vortexed. The ISOLATE II Genomic DNA spin columns were placed in a 2 mL collection tube. The samples were loaded to columns and centrifuged for 1 min at 11,000 x g. The flow-through was discarded and collection tubes were reused. The silica membrane was washed by adding 500 μL wash buffer GW1 and centrifuged for 1 min at 11,000 x g. The flow-through was discarded and collection tubes were reused. Then, 600 μL wash buffer GW2 was added and centrifuged for 1 min at 11,000 x g. The flow-through was discarded and collection tubes were reused. The silica membrane was dried by centrifugation for 1 min at 11,000 x g, to remove residual ethanol then ISOLATE II Genomic DNA spin columns were placed in a 1.5 mL collection tube. DNA elution was done by adding 100 μL preheated Elution Buffer G (70 °C) onto center of silica membrane and incubated at room temperature 1 min then the DNA collection was done by centrifuged the tubes 1 min at 11,000 x g. The DNA samples have been kept at − 20 °C for further purpose.

### Bisulfite conversion

The extracted DNA input of 200–500 ng for each sample was subjected to bisulfite treatment using the EpiJET Bisulfite Conversion Kit (Thermo Fisher Scientific, USA) according to the protocol provided by the manufacturer. The 20 μL of DNA sample containing 200–500 ng of purified genomic DNA was added into a PCR tube. The 120 μL of prepared modification reagent solution was added to 20 μL of DNA sample in a PCR tube. The sample was mixed by pipetting up and down, then centrifuged the liquid to the bottom of the tube. The PCR tubes were placed into a T100™ Thermal Cycler (Biorad, USA) and proceeded performing denaturation and bisulfate conversion of DNA: 98 °C for 10 min, 60 °C for 150 min. Then the next step was proceeded immediately by adding 400 μL of binding buffer to the DNA purification micro columns. The DNA purification micro columns were placed into the collection tube later. The converted DNA sample was loaded into the binding buffer in the columns and mixed completely by pipetting. The micro columns were placed into the collection tubes and centrifuged at 12,000 rpm for 30 s then flow-through was discarded. The micro columns were placed back into the same collection tube. The 200 μL of wash buffer was added into the micro columns and further centrifuged at 12,000 rpm for 30 s, subsequently, the flow-through was discarded. The micro-columns were placed into the same collection tube. The 200 μL of desulfonation buffer, prepared with ethanol, was added into the micro columns and left in the columns at room temperature for 20 min. The micro columns were placed into the collection tubes and then centrifuged at 12,000 rpm for 30 s. The flow-through was discarded. The micro columns were placed into the same collection tubes. The 200 μL of wash buffer, prepared with ethanol was added to the micro columns and centrifuged at 12,000 rpm for 30 s. Then, the micro columns were placed into the same collection tubes. The 200 μL of wash buffer, prepared with ethanol was added to the micro columns and centrifuged at 12,000 rpm for 60 s. Then, the columns were placed into a clean 1.5 mL microcentrifuge tubes, 10 μL of elution buffer was added to the micro columns and centrifuged at 12,000 rpm for 60 s. The converted DNA was eluted and has been ready for downstream analysis. The bisulfite treated DNA were stored at − 20 °C.

### Design of methylation and unmethylation primer and methylation-specific PCR

The selected target genes were determined which promoter were methylated. The oligonucleotides primers for MSP were designed manually and were sent to the company to be further synthesized by Integrated Device Technology (IDT), Inc., USA. (Table 2 and 3).

**Table 2:** Methylation oligonucleotide sequences and conditions for MSP

Oligonucleotides	Sequence (5’–3’)	Amplicon (bp)	Tm (°C)
*C1QBP*	FW: TAGCGGATTCGGTAGCGTAG	152	54
	RW: CCTAAAACGCCGCCGAAAAC		
*CDKN2A*	FW: TTCGAGTATTCGTTTACGGCGT	159	54
	RW: AACGCACGCGATCCGCC		
*BCAP31*	FW: TTCGGTTTTCGGTCGCGGTAT	124	55
	RW: CCAACCCGACGCGCGAAA		
*PTMS*	FW: CGTTTTATTTTTTTTTCGCGGTCG	128	54
	RW: CGACGCTACCCGACGAAAAA

**Table 3:** Unmethylation oligonucleotide sequences and conditions for MSP

Oligonucleotides	Sequence (5’–3’)	Amplicon (bp)	Tm (°C)
*C1QBP*	FW: TAGTGGATTTGGTAGTGTAG	152	48
	RW: CCTAAAACACCACCAAAAAC		
*CDKN2A*	FW: TTTGAGTATTTGTTTATGGTGT	159	46
	RW: AAACACACACAATCCACC		
*BCAP31*	FW: TTTGGTTTTTGGTTGTGGTAT	124	46
	RW: CCAACCCAACACACAAAA		
*PTMS*	FW: TGTTTTATTTTTTTTTTGTGGTTG	128	46
	RW: CAACACTACCCAACAAAAAA		

The target genes were investigated for methylation analysis. The bisulfite treated DNA was consequently used to carry out methylation-specific PCR by using methylated and unmethylated specific primers. MyTaq™ HS Mix (Bioline, UK) was used according to manufacturer’s instructions, following standard MyTaq™ HS Mix protocol. The total volume of PCR reaction was 25 μL (MyTaq HS Mix, 2 × 12.5 μL, 10 μM forward primer 0.5 μL, 10 μM reverse primer 0.5 μL, template DNA 200 ng and PCR-grade water (Bioline, UK) up to 25 μL) The qPCR parameters were adjusted properly according to the manufacturer’s protocol (initial denaturation 95 °C 1 min, denaturation 95 °C 30 s, annealing temperature as described for each gene primer set 45 s and extension 72 °C 30 s for total 40 cycles, dissociation step is depended on instrument guidelines). EpiTect PCR Control DNA Set (Qiagen, USA) contained methylated human control DNA (bisulfite 100 μL converted) 10 ng/μL, unmethylated human control DNA (bisulfite 100 μL converted)10 ng/μL and unmethylated human control DNA 10 ng/μL were used as internal control. PCR products were observed by 1% agarose (Bio Basic, Canada) by gel electrophoresis using GelDoc™ XR^+^ (Biorad, USA). The methylated and unmethylated band intensities of each sample were visualized and measured using GelQuant.NET Software (Biochem Lab Solutions, CA, USA).

### Statistical data analysis

All data with average of triplicates was reported. Paired sample *t*-tests was applied for two groups comparison in each experiment, *p* ≤ 0.05 was indicated statistical significance.

## Results

### E7 protein expression in a transfected HEK293 cell line

**Fig. 1:**
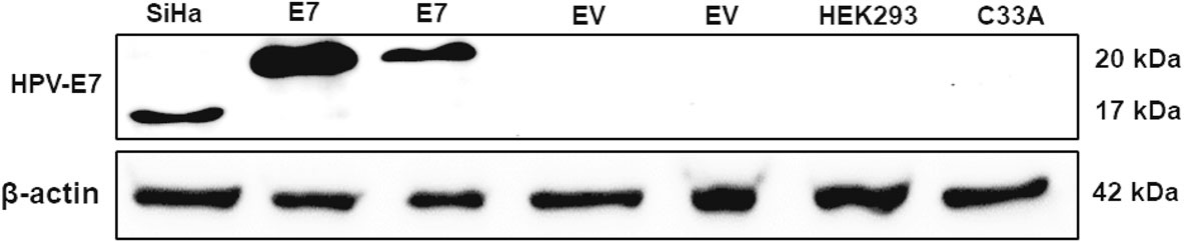
Western blot analysis of expression of E7 in transfected HEK293. HEK293 was transfected with recombinant E7 plasmid and empty vector as control. SiHa was used as positive control. The samples were run SDS-PAGE and blotted with anti-HPV type 16 E7 antibody. E7 bands were showed approximately at 17 and 20 kDa in SiHa and E7-transfected HEK293, respectively. No positive bands were observed in negative control (EV-transfected and untransfected HEK293, C33A (HPV negative cell line), respectively), Western blot analysis of β actin as internal control were showed at 42 kDa

To examine the transfection efficiency of a recombinant histidine-tagged E7 construct in HEK293 cells, the protein expression in lysates from E7-transfected, empty vector (EV) transfected and untransfected HEK293 cells was examined by immunoblot using anti-E7 antibody. As shown in results from immunoblotting in Fig. 1, E7 bands were observed at 20 kDa in E7- transfected HEK293 samples and at 17 kDa in SiHa. E7 protein bands were not observed in EV-transfected and untransfected HEK293 and C33A lysates. β-actin was used as internal control, which presented a 42 kDa band.

### E7-induced alteration of HEK293 proteome

**Fig. 2:**
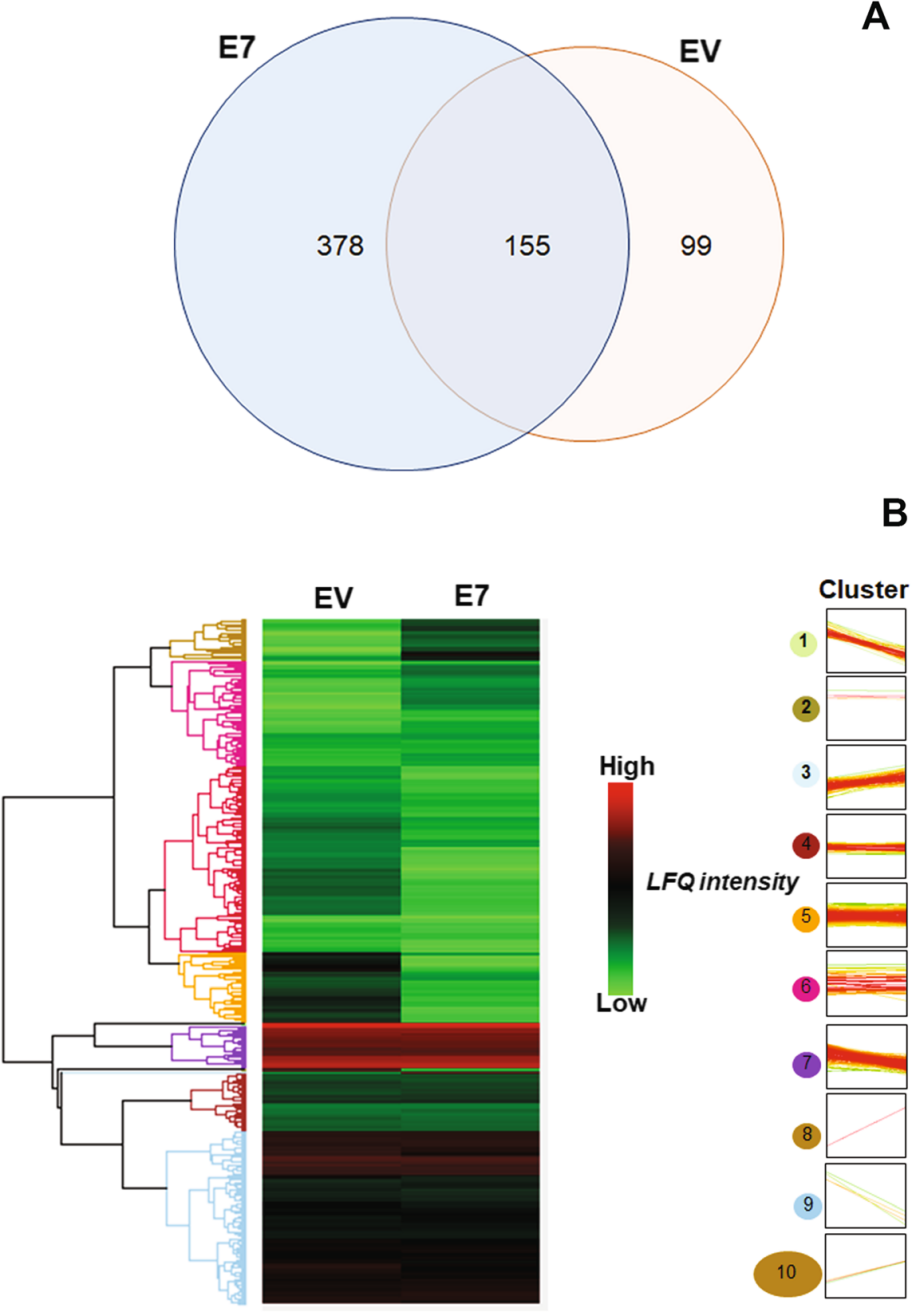
Venn diagram of number of identified proteins and hierarchical clustering heatmap by Perseus. EV and E7 transfected HEK293 protein samples were analyzed and identified with Maxquant, the number of identified proteins in E7-transfected HEK293 was higher than in EV-transfected HEK293 **(**a**)**. Expression of proteins in EV and E7-transfected HEK293 were clustered as 10 groups, which cluster 1, 7 and 9 showed down-regulated pattern in E7-transfected HEK293 (b)

**Fig. 3:**
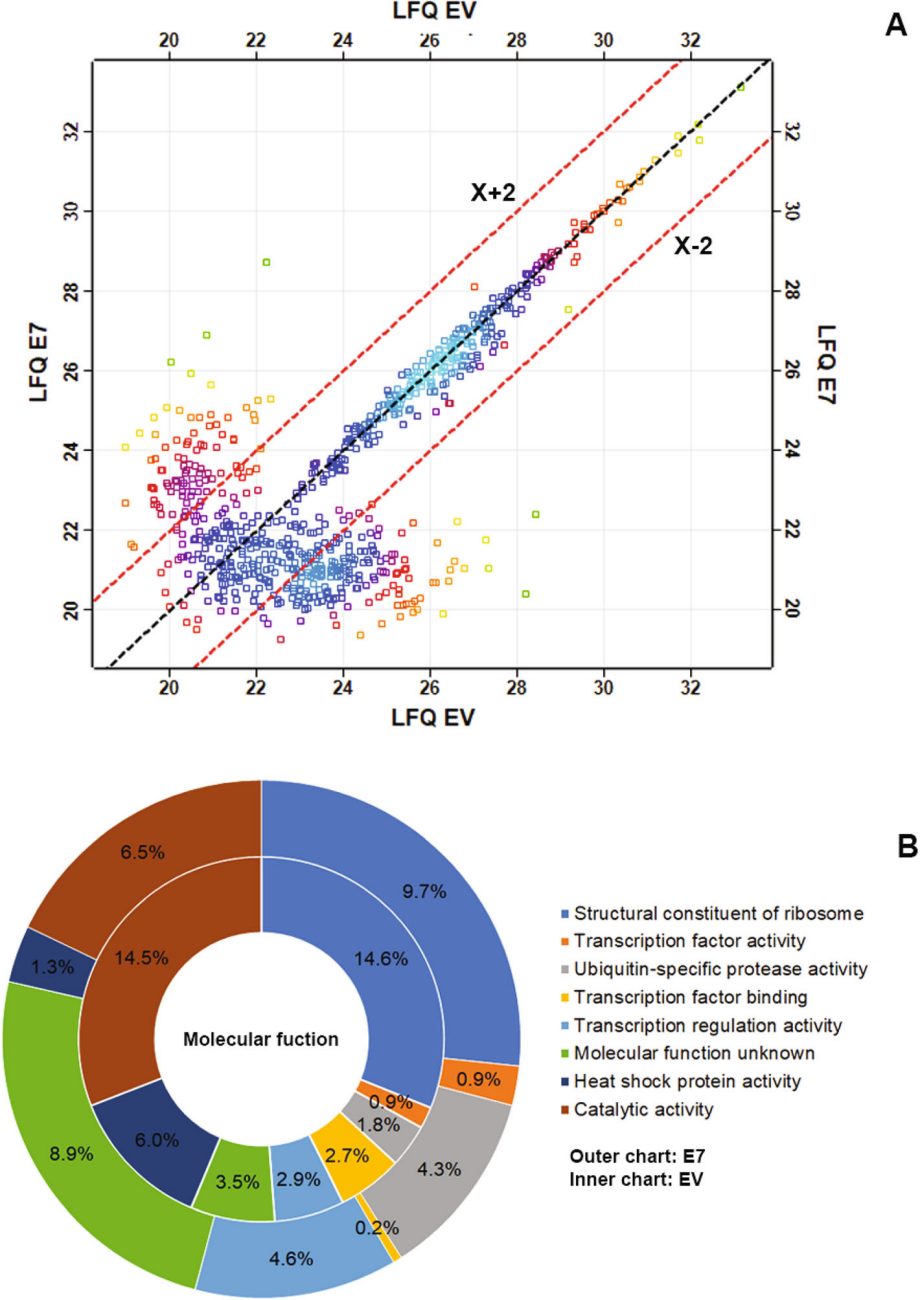
Total protein scatter plot generated by Maxquant and protein categorization based on Gene Ontology. The total proteins were categorized by molecular function and biological pathway and it demonstrated that majority of proteins was classified as RNA binding, metabolism of RNA and proteins **(a)**. Scattering plot of expressed proteins, the proteins under line showing down regulation and were selected to annotated protein function based on cancer pathways (**b**)

We further explored E7-driven host proteome changes, comparing E7- and EV-transfected HEK293 cell proteomes. The Maxquant database search algorithm providing a greater number (Additional file 1: Figure S1 and Additional file 2: Table S1 and Additional file 3: Table S2) compared with SEQUEST (passed FDR ≤ 0.05). Several proteins were found at lower levels in E7-transfected HEK293 than in EV-transfected HEK293 (Fig. 2). Proteome-wide expression analysis showed differential expression after hierarchical heatmap clustering (Fig. 2b). We focused on down-regulated proteins (potentially resulting from gene silencing associated with aberrant host DNA methylation). According to hierarchical clustering, protein expression pattern was clustered into 10 groups. Clusters 1 and 7 contained down-regulated proteins relative to other clusters. We also analyzed down-regulated proteins in a scatter plot (Fig. 3a). Proteins with lower than two-fold down-regulated expression (as defined by Maxquant transformed label-free-quantification) were selected for further function annotation. From this information, we constructed a pathway enrichment heatmap for nominating candidate genes relevant to cancer pathways.

### Protein functional enrichment analysis

Identified proteins from E7- and EV-transfected cells were analyzed using FunRich protein annotation analysis, which combines gene enrichment with protein quantity data (Fig. 3b). Major differences in functional enrichment between E7*-* and EV-transfected cells are observed for several protein classes. In E7-transfected HEK293, proteins were increased related to; ubiquitin-specific protease activity (4.3%), transcription factor activity (2.4%), transcription factor binding (0.2%) and transcription regulator activity (4.6%). Conversely, protein functions for; the structural constituents of ribosomes (9.7%), catalytic activity (6.5%) and heat shock protein activity (1.3%) were decreased. Another 8.9% of major differences were attributed to proteins of unknown function.

### Down-regulated proteins preferentially expressed in particular gene ontology (GO) annotation pathways

**Fig. 4:**
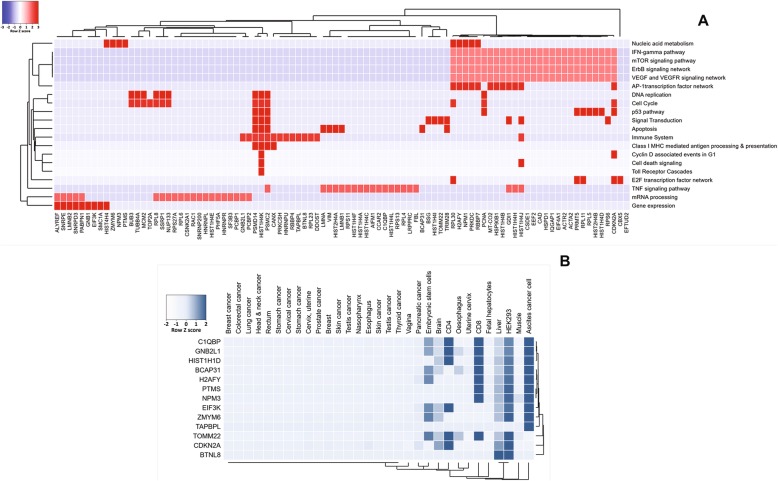
Pathway enrichment of overall identified proteins, enrichment heatmap of down-regulated proteins and expression mapping of gene candidates heatmap. Down-regulated proteins were annotated against cancer pathways for seeking for gene candidates to further mRNA expression and DNA methylation analysis (**a**). Protein expression across several cell types and tissues, gene candidates were annotated against protein expression pattern from Human protein atlas database. Apparently, selected proteins relatively less express in cancer cell type and tissues (**b**)

To identify host gene candidates for specific E7-mediated DNA promoter hypermethylation, we mapped down-regulated proteins against several cancer-associated pathways. The protein annotation heatmap (Fig. 4a) indicated the function of E7-mediated down-regulated proteins, which mostly mapped to the interferon gamma pathway, the mTOR signaling pathway, the ErbB receptor signaling network and VEGF/VEGFR signaling network. In addition, there were a smaller number of proteins mapped to other major cancer-associated pathways such as immune systems and p53 pathway. We selected indicative candidate proteins for mRNA transcript level and promotor DNA methylation status analysis. Candidates were selected that were distributed in cancer associated pathways related to host immunity, the cell cycle and HPV, that have been described in the literature. Thirteen gene candidates were selected: Q07021 (*C1QBP*), P42771 (*CDKN2A*), O95789 (*ZMYM6*), P51572 (*BCAP31*), P16402 (*HIST1H1D*), O75367 (*H2AFY*), O75607 (*NPM3*), P63244 (*RACK1*), P20962 (*PTMS*), Q9BX59 (*TAPBL)*, Q6UX41 (*BTNL8*), Q9UBQ5 (*EIF3K*) and Q9NS69 (*TOMM22*). Expression data of gene candidates from the Human Protein Atlas database followed by hierarchical clustering by Funrich analysis showed mostly down-regulation in cancer tissues and cell lines compared with normal tissues and cell lines (Fig. 4b).

### Determination of transcript and protein abundance correlation for gene candidates

Although gene candidates were selected from down-regulated proteins associated with cancer pathways, protein abundances are often not correlated with mRNA transcript abundances. Therefore, we measured the mRNA expression of gene candidates using qPCR and compared transcript expression in *E7*-transfected HEK293 cells to expression in SiHa cells.

**Fig. 5:**
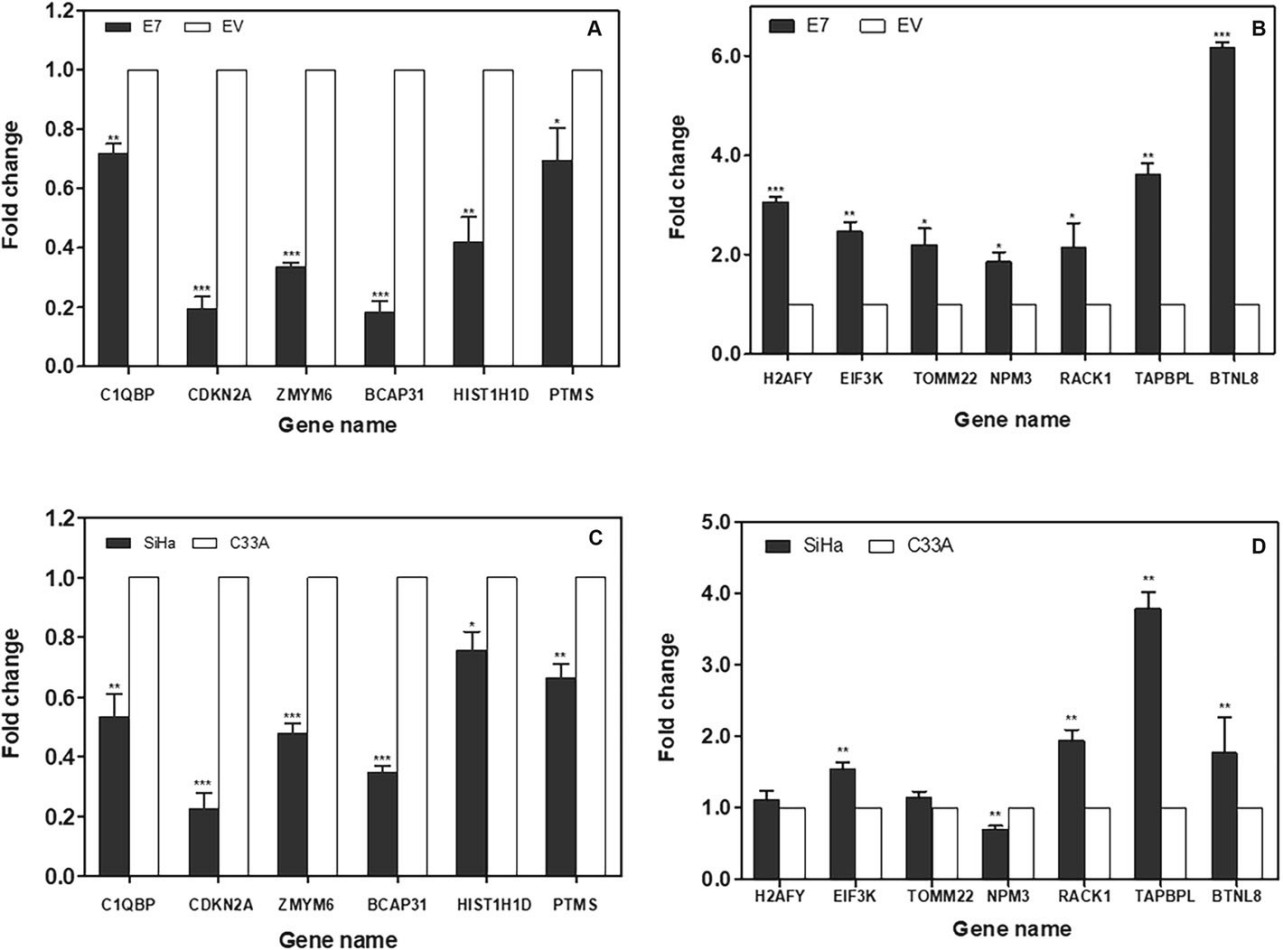
Gene expression at mRNA level of selected gene candidates. Selected gene candidates were measured expression at mRNA level in E7-transfected HEK293 with RT-qPCR compared to expression in SiHa. The y-axis represented for fold change (GAPDH was used as reference gene). Expression of selected gene candidates in E7-transfected HEK293 from proteomic data were apparently down-regulated (*left panel*) and up-regulated (*right panel*) with correspondence to the expression in SiHa (*, ** and *** are statistical significance *p*-value ≤0.05,0.01 and 0.001, respectively)

Six gene candidates were found to be significantly down-regulated in E7-transfected HEK 293 cells compared with control: *C1QBP* (*p* = 0.0031), *CDKN2A* (*p* = 0.0003), *ZMYM6* (*p* < 0.0001), *BCAP31* (*p* = 0.0002), *HIST1H1D* (*p* = 0.0067) and *PTMS* (*p* = 0.0222). Interestingly, these genes comprised 6 of the 7 genes that are downregulated in the control cervical cancer cell lines (SiHa and C33A): *C1QBP* (*p* = 0.0092), *CDKN2A* (*p* = 0.0008), *ZMYM6* (*p* = 0.0006), *BCAP31* (*p* < 0.0001), *HIST1H1D* (*p* = 0.0294), *NPM3* (*p* = 0.0083) and *PTMS* (*p* = 0.0053) as shown in Fig. 5. In contrast, we found that seven of the gene candidates were significantly up-regulated in E7*-*transfected HEK 293 cells compared with control: *H2AFY* (*p* = 0.0003), *EIF3K* (*p* = 0.0046), *TOMM22* (*p* = 0.0366), *NPM3* (*p* = 0.0183), *RACK1* (*p* = 0.0297), *TAPBPL* (*p* = 0.0012) and *BTNL8* (*p* < 0.0001). Four of these genes were also significantly up-regulated in cervical cancer cell lines (SiHa and C33A):*EIF3K* (*p* = 0.0092), *RACK1* (*p* = 0.0086), *TAPBPL* (*p* = 0.0011) and *BTNL8* (*p* = 0.0012). However, two genes were not significantly up-regulated: *H2AFY* (*p* = 0.4522) and *TOMM22* (*p* = 0.1847) as shown in Fig. 5.

### HPV-E7 increased methylation of *C1QBP*, *BCAP31*, *CDKN2A* and *PTMS* gene promoter methylation in HPV positive cervical cell lines

**Fig. 6:**
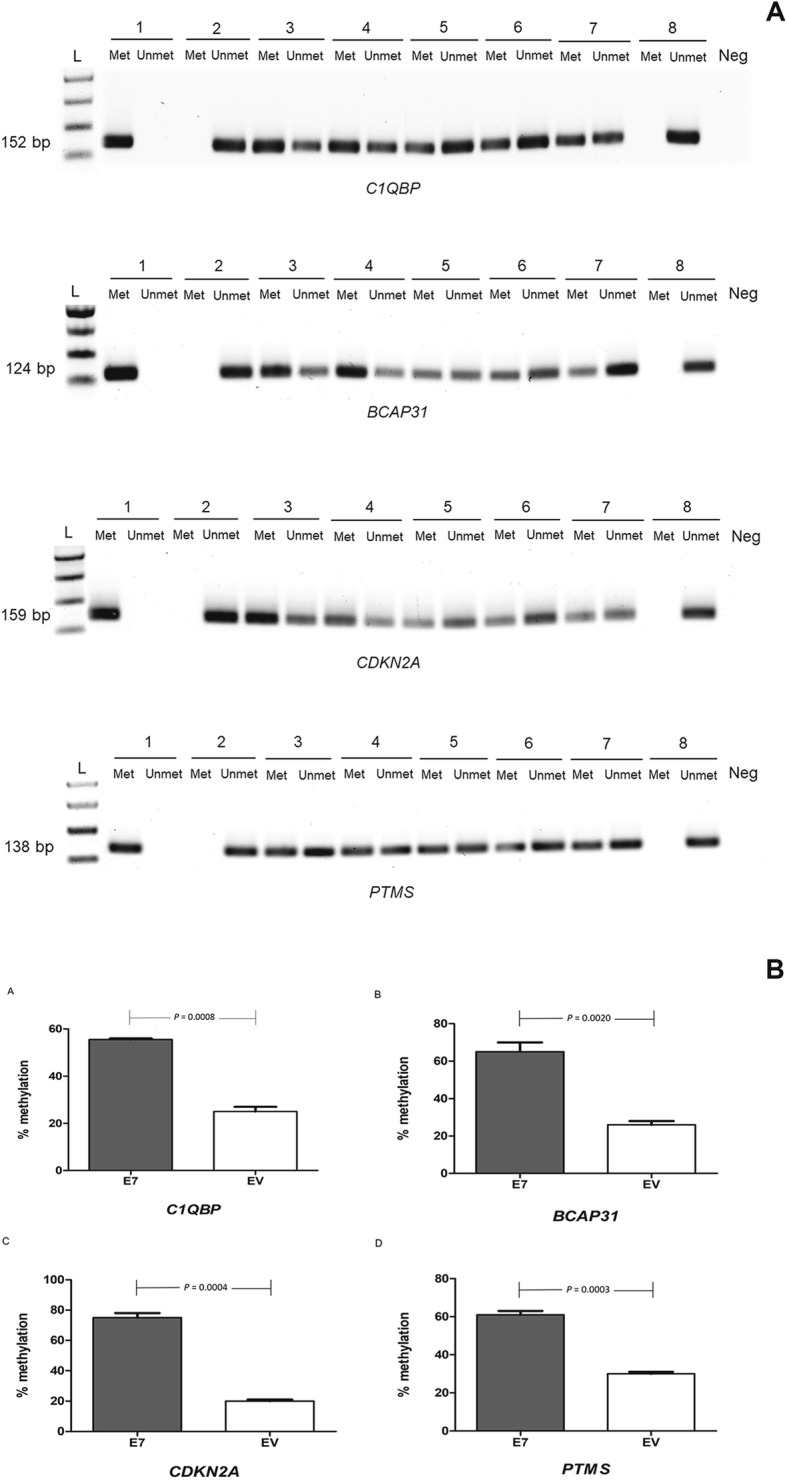
Hypermethylation of down-regulated gene expression. Agarose gel electrophoresis of promoter methylation of selected down-regulated gene candidates, *C1QBP*, *BCAP31*, *CDKN2A* and *PTMS*, which PCR product bands were showed at 152, 124, 159 and 138 bp, respectively (PCR products were amplified with met or unmet primers, which *lane 1–2*: methylated human control DNA, *lane 3–4*: unmethylated human control, *lane 5–6*: SiHa cell line, *lane 7–8*: E7-transfected HEK293, *lane 9–10*: EV-transfected HEK293, *lane 11–12*: untransfected HEK293, *lane 13–14*: C33A, *lane 15–16*: unmethylated human control DNA and “Neg” referred to negative control) (**a**). Percentage of methylation of gene candidate promotors. The DNA methylation of promoter of selected gene candidates (*C1QBP*, *BCAP31*, *CDKN2A* and *PTMS*) in E7–transfected HEK293 were significantly increased compared to EV-transfected HEK293, *p*-values of each experiment were shown above between bars (**b**)

**Fig. 7:**
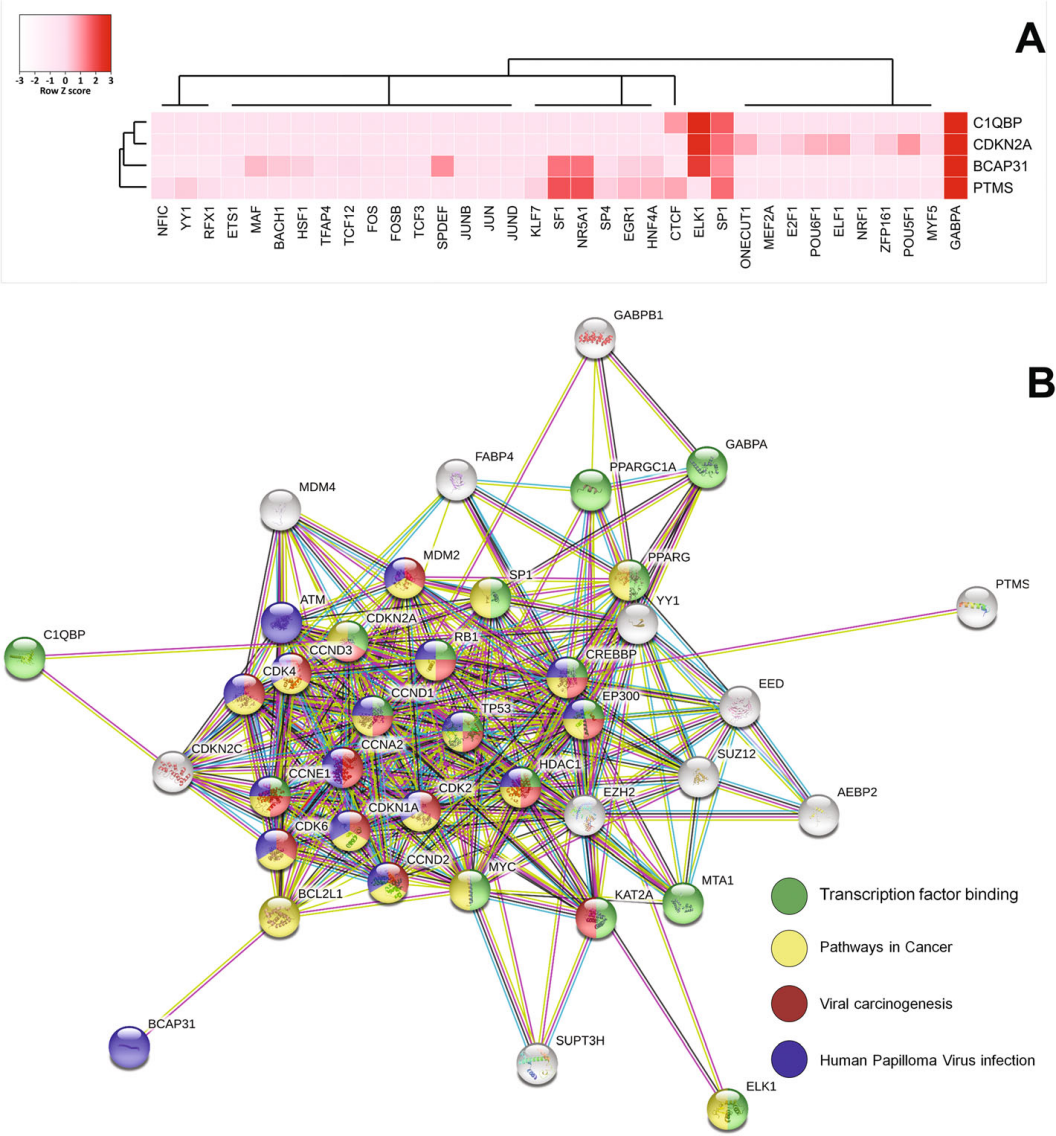
Prediction of putative regulatory transcription factors and STRING database protein network analysis. Heatmap of regulatory transcription factors of hypermethylated gene candidate promoters, the heatmap demonstrated that GABPA, SP1 and ELK1 were putative transcription factors that possibly participated in DNA methylation (**a**). STRING database protein network analysis mapping were performed for seeking interacting proteins that presumably associate with transcription factors contributing E7-mediated hypermethylation. The STRING network analysis indicated that E7-mediated hypermethylated genes and putative transcription factors were mostly relevant to cell cycle, viral carcinogen and HPV infection (**b**)

We selected four down-regulated genes in E7-transfected HEK293 cells that were also downregulated in SiHa cells (*C1QBP*, *BCAP31*, *CDKN2A* and *PTMS*) and assessed methylation of the relevant gene promoters in E7- and *E*V-transfected HEK293, untransfected HEK 293 and SiHa cells. Methylated DNA bands for *C1QBP*, *BCAP31*, *CDKN2A* and *PTMS* (at 152, 124, 159 and 128 bp, respectively) were greatly increased in SiHa and *E7*-transfected HEK293 cells compared with the negative controls of untransfected HEK293, EV-transfected HEK293 and C33A cells (Fig. 6a). A methylated human control DNA (bisulfite converted) showed positive bands in these methylation primer sets, while unmethylated human control DNA did not show positive bands. The Fig. 6b demonstrated the percentage *C1QBP* promoter hypermethylation in E7- and EV-transfected HEK293 was 55.5 and 25% (*p* = 0.0008), respectively; for *BCAP31* the corresponding figures were 65 and 26% (*p* = 0.0020), respectively; for *CDKN2A,* 75 and 20% (*p* = 0.0004), respectively (Fig. 7c); for *PTMS,* 61 and 30% (*p* = 0.0003), respectively.

### Prediction of putative regulatory transcription factors of E7-mediated host specific promoter hypermethylation

We conducted analysis to determine potential host transcription factors interacting with E7 to implement specific gene-promoter hypermethylation. The heatmap in Fig. 7a identified GA-binding protein alpha chain (GABPA), specificity protein 1 (SP1) and ETS Like protein-1 (ELK1) as possible transcription factors for *C1QBP*, *BCAP31*, *CDKN2A* and *PTMS*. We used the STRING database to identify potential interacting protein partners with E7 and these host transcription factors. Protein networking prediction by STRING database demonstrated GABPA interacts to SP1, which interact to E7 interacting proteins i.e. p53 and Rb1 (Fig. 7b). Thereby, apparently, GABPA, SP1 and ELK1 potentially interact a group of proteins that function in HPV associated pathways as well as transcription factors binding, in which CDKN2A also the key protein in these pathways as well. Moreover, C1QBP, BCAP31 and PTMS protein were predicted as their interactor pathways in cancer.

## Discussion

### The expression of E7 altered the proteome of HEK293 cells

Although it is commonly known that HPV proteins E6 and E7 play crucial and interlinked roles in cancerous tissue transformation, the effects of E7 alone in tumorigenesis are not fully understood. Particularly with respect to the links between E7-driven DNA methylation of specific gene promotors at early cancer stages and host proteome aberrations. We employed HEK293 as naïve epithelial cell model, previously unexposed to HPV. Despite HEK293 not being a cervical cell type, a previous study reported that this line can be used for studying this cancer [22]. In addition, HEK293 cells generally have high transfection efficiency, and thus we could be confident that E7 transfection would reliably influence the proteome. Our proteomic analysis revealed that E7 likely influenced protein levels through transcription factor/regulator binding and modulation, while reducing ubiquitin-specific protease activity and heat shock protein activity. Gene enrichment analysis implied that E7 also modifies host transcription from an epigenetic level. DNA methylation is a commonly employed viral mechanism to suppress specific protein expression in the host proteome. Here, we chose down-regulated proteins from proteomic analysis, that play crucial roles in cancer-associated pathways, for our analysis of the corresponding hypermethylated gene candidates. Comparing the selected down-regulated proteins with the expression in other types of cancers in human atlas database, these down-regulated proteins mostly express less compared to normal tissues. Therefore, this further convinced us that E7 influenced the proteins have been identified that down-regulation in cancer tissues. In which, the transcript abundances of these proteins were measured to strengthen the link between E7-mediated protein downregulation and corresponding gene hypermethylation.

### Expression of candidate mRNA transcripts compared with corresponding protein levels and E7-mediated DNA methylation of gene candidates

We measured the relative abundance of mRNA transcripts for the 13 gene candidates by qPCR, to determine the correlation to the corresponding protein levels assessed in proteomics data. Data were compared with expression in the SiHa cell line.

Six genes out of the 13 candidates were down-regulated at the transcriptional level (*C1QBP*, *CDKN2A*, *ZMYM6*, *BCAP31*, *HIST1H1D* and *PTMS*), while four were upregulated (*EIF3K*, *RACK1*, *TAPBPL* and *BTNL8),* however, proteomic data demonstrated all their corresponding protein abundances were down-regulated, In SiHa cells, the 13 gene candidates were expressed in a corresponding pattern to the transfected HEK293 cells, suggesting that E7 drives the expression pattern of these genes candidates in same manner that HPV drives their expression in cancer. However, SiHa also expresses E6 making it inconclusive that E7 alone is able to drive tumorigenesis through dysregulation of those 13 genes. Nevertheless, it is evident that the expression of those 13 genes is influenced by E7 from proteomic and qPCR results in *E7*-transfected HEK293 cells. In addition, four down-regulated genes in our transformed HEK293 cells, with corresponding expression in SiHa and linked to HPV-altered signaling pathways (*C1QBP*, *CDKN2A*, *BCAP31* and *PTMS*), were selected for methylation analysis of their promotors. Interestingly, we found all their promoters were hypermethylated in both E7-transfected HEK293 and SiHa, whereas methylation of those genes was lower in *EV*-transfected HEK293 and C33A. This result implies that E7 modulates *C1QBP, CDKN2A, BACAP31* and *PTMS* levels through DNA methylation and that this modulation could play a role in precancerous progression, though it should be noted that methylation of those genes in the cancerous C33A cell line was low.

Here, we postulated E7 binds to host transcription factors to form a complex with DNMT1 and binds to a specific genome region via transcription factors. Subsequently, DNMT1, which has enhanced methylase activity when bound to E7, methylates the specific genome region. We constructed a predictive heatmap to ascertain putative regulatory transcription factors for *C1QBP, CDKN2A, BCAP31* and *PTMS*. From our analysis, GABPA demonstrated the highest possibility for regulating the four gene candidates followed by ELK1 and SP1. GABPA, SP1 and ELK1 have common cis-regulatory elements and probably form a complex as a result of E7 activation [23]. A previous study has mentioned that GABPA likely interacts with SP1 and plays a part in regulating broad biological processes such as; embryonic development, cell differentiation, mitochondrial biogenesis and the cell cycle [24]. GABPA and SP1 have both been linked to HSV type 16-mediated transcription regulation, and moreover, are able to facilitate DNA methylation of the E7 upregulated genes identified here [25, 26]. GABPA belongs to the EST transcription factor family, which are able to bind to several cis-regulatory elements, including *C1QBP*, *CDKN2A*, *BCAP31* and *PTMS*. Suppression of these genes by hypermethylation, and a reduction in their protein abundance, possibly promotes carcinogenesis. Notably cancerous cells often express *CDKN2A* at low levels mostly, in which through gene methylation [27, 28].

### Biological function of hypermethylated gene promotors

The effects of HPV-E7 oncoprotein on host gene promoter methylation have been extensively studied. For example, E7 affects the methylation of E-cadherin, involved in Langerhans cell delivery through the mucosal epithelium layer, to instigate an immune response to HPV infection [29–33]. E7 possibly also drives hypermethylation of the cyclin A1 (*CCNA1*) promoter, resulting in decreased cyclin A1 mRNA expression and uses the same mechanism to repress *HLA-E* expression, downregulating MHC class I, the antigen presenting complex on natural killer and CD8^+^ T cells [14–16].

*C1QBP* encodes the multi-functional protein complement component 1Q subcomponent binding protein (C1QBP), extensively found in various tissues and cell types. C1QBP is mainly found in mitochondria but also, occasionally, in the cytosol, cell surface and nucleus of many cell types [34–36]. Several studies have revealed the functions of C1QBP in tumorigenesis are varied: for instance, C1QBP maintains oxidative phosphorylation for tumor cells, enhances cell chemotaxis and metastasis in breast cancer and suppresses the Y box binding protein 1 in renal cell carcinoma [37–42]. Additionally, suppression of C1QBP by HPV type 16, dramatically induced cancer cell immune evasion [42, 43]. *C1QBP* promoter methylation could impede the host immune response by modifying interleukin 6 production and dendritic cell metabolism and maturation; activity important in cervical cancer cell progression [44, 45]. However, to our knowledge, no report has mentioned that E7 mediates suppression of C1QBP. Our findings here demonstrate that *C1QBP* promoter methylation is significantly increased in *E7*-transfected HEK293 compared with control and is a subject of E7-targeted methylation.

*BCAP31*, coding for B-cell receptor-associated protein 31 (BCAP31), is an endoplasmic reticulum membrane protein that acts as a molecular chaperone. It is associated with apoptosis regulation through the caspase 8 pathway, protein transport and degradation, promoting the vesicular transport of transmembrane proteins (such as MHC 1, CD11b/CD18, cytochrome 450 and cellubrevin) and B- and T-cell activation [46–53]. Enhanced BCAP31 expression has been observed to decrease survival of non-small-cell lung carcinoma cells [54]. However, overexpression in gastric cancer stimulated proteasome degradation of p27kip1, leading to cell proliferation [55]. Interestingly, from a previous study, BCAP31 has been established as a key target of high-risk HPV E5 protein, modulating cancer cell differentiation in cervical cancer [56]. To our knowledge, the effect of E7 on BCAP31 had not been identified. Here, we found that BCAP31 protein and *BCAP31* gene expression levels were decreased in E7-transfected HEK293 compared with control and that promoter methylation status in E7-transfected cells was markedly increased. This result suggests that E7, in addition to E5, contributes to HPV pathogenesis by targeting BCAP31 through gene silencing.

*CDKN2A*, encodes the p16^INK4a^ tumor suppressor protein of the cyclin dependent kinase inhibitor family, which functions by blocking cyclin dependent kinase 4/6 interaction with cyclin D1 in the cell cycle. This event can heavily suppress phosphorylation of pRb, prohibiting cell cycle progression from G1 to S phase [57]. Inactivation of p16^INK4a^ can strongly support cell proliferation in various types of carcinomas, including gastric cancer, glioma, bladder cancer, breast cancer and head and neck cancer [58, 59]. Dysregulation of the *CDKN2A* gene has also been a prominent observation in oral squamous cell carcinoma [60–63]. Suppression of *CDKN2A* gene expression generally occurs by aberrant methylation of its promoter, and it also plays a crucial role during neoplastic progression in cervical cancer [64, 65]. The methylation of *CDKN2A* promoter may occur between low-grade and high-grade cervical dysplasia and is common in invasive carcinoma which express the E7 oncoprotein [66, 67]. Here, we observed that promoter methylation of *CDKN2A* was significantly increased in E7- transfected HEK293 compared with control. This result is consistent with protein and gene expression level in a recent study identifying *CDKN2A* promoter methylation as a diagnostic biomarker of E7-expressing cervical dysplasia [68].

*PTMS* encodes for parathymosin alpha (PTMS), an immunoregulatory protein which elicits interleukin 2 expression in human lymphocytes, and cooperates with αB-interferon in stimulating natural killer cell activity and involved in the early DNA replication and apoptosis process [69–72]. However, PTMS has not been studied in cervical cancer intensively, particularly HPV-mediated gene methylation. In addition, our results show high methylation status for the PTMS promoter in *E7*- transfected HEK293, compared with control, in agreement with low expression levels of protein and mRNA. These results suggest that E7-targeted methylation is responsible for PTMS activities in immune regulation and apoptosis activation enhancement by HPV. However, in summary, this study still requires further research on to validate the interaction of gene candidates, transcription factors and how E7 interacts with specific transcription factors by other experiments such as chromatin immunoprecipitation and further gene methylation in clinical samples.

## Conclusion

This study has identified proteins downregulated by the HPV E7 oncoprotein and linked them to targeted promoter hypermethylation of their corresponding genes. In particular, four genes, *C1QBP, BCAP31, CDKN2A* and *PTMS* are identified. These genes may serve as potential biomarkers for HPV infection or be target genes for cervical cancer therapeutics. Our findings are a primary report, and further investigations into the roles of these genes in HPV-mediated cervical cancer progression will be important to improve cervical cancer detection and treatment.

## Supplementary information

**Additional file 1 : Figure S1.** Venn diagram of comparison of number of identified proteins with SEQUEST (Proteome Discoverer 2.2) and Maxquant database searching algorithm. The Venn diagram showed that Maxquant identified more proteins compared to SEQUEST but are mostly common. The commonly identified proteins by two algorithms were selected to further transcript abundance determination.

**Additional file 2 : Table S1.** List of total identified proteins of E7-transfected HEK293 by Proteome Discover.

**Additional file 3 : Table S2.** List of total identified proteins of E7-transfected HEK293 by Maxquant.

## Data Availability

Please contact author for data requests.

## References

[1] WHO/ICO Information Center of HPV and Cervical Cancer (HPV Information Center). Human Papillomavirus and Related Cancers in the World. In: Summary Report 2017. http://www.who.int/hpvcentre/en. Accessed 18 Jul 2017.

[2] Pinidis P, Tsikouras P, Iatrakis G, Zervoudis S, Koukouli Z, Bothou A (2016). Human papilloma Virus’ life cycle and carcinogenesis. Medica (Buchar).

[3] Pan Yunbao, Liu Guohong, Zhou Fuling, Su Bojin, Li Yirong (2017). DNA methylation profiles in cancer diagnosis and therapeutics. Clinical and Experimental Medicine.

[4] Hamborsky J, Kroger A, Wolf S, Epidemiology and Prevention of Vaccine-Preventable Diseases. 13^th^ ed. Centers for Disease Control and Prevention. The Pinkbook. 2016;(11):175–86.

[5] National Cancer Institute (NCI). Fact sheet: HPV and cancer. https://www.cancer.gov/about-cancer/causesprevention/risk/infectious-agents/hpv-fact-sheet. Accessed 18 Jul 2017.

[6] McMurray HR, Nguyen D, Westbrook TF, McAnce DJ (2001). Biology of human papillomaviruses. Int J Exp Pathol.

[7] Christmann M, Kaina B (2019). Epigenetic regulation of DNA repair genes and implications for tumor therapy. Mutat Res.

[8] Au Yeung CL, Tsang WP, Tsang TY, Co NN, Yau PL, Kwok TT (2010). HPV-16 E6 upregulation of DNMT1 through repression of tumor suppressor p53. Oncol Rep.

[9] Cutts FT, Franceschi S, Goldie S, Castellsague X, de Sanjose S, Garnett G (2007). Human papillomavirus and HPV vaccines: a review. Bull World Health Organ.

[10] Lee LY, Garland SM (2017). Human papillomavirus vaccination: the population impact. F1000Res.

[11] Sahasrabuddhe VV, Luhn P, Wentzensen N (2011). Human papillomavirus and cervical cancer: biomarkers for improved prevention efforts. Future Microbiol.

[12] Burgers WA, Blanchon L, Pradhan S, de Launoit Y, Kouzarides T, Fuks F (2007). Viral oncoproteins target the DNA methyltransferases. Oncogene..

[13] Virani S, Colacino JA, Kim JH, Rozek LS (2012). Cancer epigenetics: a brief review. ILAR J.

[14] Chalertpet K, Pakdeechaidan W, Patel V, Mutirangura A, Yanatatsaneejit P (2015). Human papillomavirus type 16 E7 oncoprotein mediates CCNA1 promoter methylation. Cancer Sci.

[15] Cicchini L, Blumhagen RZ, Westrich JA, Myers ME, Warren CJ, Siska C (2017). High-risk human papillomavirus E7 alters host DNA Methylome and represses HLA-E expression in human keratinocytes. Sci Rep.

[16] Yang H-J (2013). Aberrant DNA methylation in cervical carcinogenesis. Chinese J Cancer.

[17] Cuzick J, Bergeron C, von Knebel DM, Gravitt P, Jeronimo J, Lorincz AT (2012). New technologies and procedures for cervical cancer screening. Vaccine..

[18] Mersakova S, Nachajova M, Szepe P, Kasajova PS, Halasova E (2016). DNA methylation and detection of cervical cancer and precancerous lesions using molecular methods. Tumour Biol.

[19] Milutin Gašperov Nina, Sabol Ivan, Planinić Pavao, Grubišić Goran, Fistonić Ivan, Ćorušić Ante, Grce Magdalena (2015). Methylated Host Cell Gene Promoters and Human Papillomavirus Type 16 and 18 Predicting Cervical Lesions and Cancer. PLOS ONE.

[20] Shanmugasundaram S, You J. Targeting Persistent Human Papillomavirus Infection. Viruses. 2017;9(8):E229. 10.3390/v9080229 PMC5580486 28820433

[21] Delpu Y, Cordelier P, Cho WC, Torrisani J (2013). DNA methylation and cancer diagnosis. Int J Mol Sci.

[22] Dumont J, Euwart D, Mei B, Estes S, Kshirsagar R (2016). Human cell lines for biopharmaceutical manufacturing: history, status, and future perspectives. Crit Rev Biotechnol.

[23] Zhang L, Yu H, Wang P, Ding Q, Wang Z (2013). Screening of transcription factors with transcriptional initiation activity. Gene..

[24] Yang ZF, Mott S, Rosmarin AG (2007). The Ets transcription factor GABP is required for cell-cycle progression. Nat Cell Biol.

[25] Vogel JL, Kristie TM (2013). The dynamics of HCF-1 modulation of herpes simplex virus chromatin during initiation of infection. Viruses..

[26] Goto Shunya, Takahashi Masashi, Yasutsune Narumi, Inayama Sumiki, Kato Dai, Fukuoka Masashi, Kashiwaba Shu-ichiro, Murakami Yasufumi (2019). Identification of GA-Binding Protein Transcription Factor Alpha Subunit (GABPA) as a Novel Bookmarking Factor. International Journal of Molecular Sciences.

[27] Li J, Zhou C, Zhou H, Bao T, Gao T, Jaing X, Ye M (2016). The association between methylated CDKN2A and cervical carcinogenesis, and its diagnostic value in cervical cancer: a meta-analysis. Ther Clin Manag.

[28] Zhou C, Shen Z, Ye D, Li Q, Deng H, Liu H, Li J (2018). The association and clinical significance of CDKN2A promoter methylation in head and neck squamous cell carcinoma: a meta-analysis. Cell Physiol Biochem.

[29] Gao P, Zheng J (2010). High-risk HPV E5-induced cell fusion: a critical initiating event in the early stage of HPV-associated cervical cancer. Virol J.

[30] Schiller JT, Day PM, Kines RC (2010). Current understanding of the mechanism of HPV infection. Gynecol Oncol.

[31] Sen Shrinka, Mandal Paramita, Bhattacharya Amrapali, Kundu Sudip, Roy Chowdhury Rahul, Mondal Nidhu Ranjan, Chatterjee Tanmay, Chakravarty Biman, Roy Sudipta, Sengupta Sharmila (2017). Impact of viral and host DNA methylations on HPV16-related cervical cancer pathogenesis. Tumor Biology.

[32] Jiménez-Wences H, Peralta-Zaragoza O, Fernández-Tilapa G (2014). Human papilloma virus, DNA methylation and microRNA expression in cervical cancer. Oncol Rep.

[33] Durzynska J, Lesniewicz K, Poreba E (2017). Human papillomaviruses in epigenetic regulations. Mutation Res Rev Mutat Res.

[34] Soltys BJ, Kang D, Gupta RS (2000). Localization of P32 protein (gC1q-R) in mitochondria and at specific extramitochondrial locations in normal tissues. Histochem Cell Biol.

[35] Fogal V, Zhang L, Krajewski S, Ruoslahti E (2008). Mitochondrial/cell-surface protein p32/gC1qR as a molecular target in tumor cells and tumor stroma. Cancer Res.

[36] Peerschke EI, Ghebrehiwet B (2007). The contribution of gC1qR/p33 in infection and inflammation. Immunobiology.

[37] Fogal V, Richardson AD, Karmali PP, Scheffler IE, Smith JW, Ruoslahti E (2010). Mitochondrial p32 protein is a critical regulator of tumor metabolism via maintenance of oxidativephosphorylation. Mol Cell Biol.

[38] Zhang X, Zhang F, Guo L, Wang Y, Zhang P, Wang R, Zhang N, Chen R (2013). Interactome analysis reveals that C1QBP is associated with cancer cell chemotaxis and metastasis. Mol Cell Proteomics.

[39] Wang Y, Yue D, Xiao M, Qi C, Chen Y, Sun D (2015). C1QBP negatively regulates the activation of oncoprotein YBX1 in the renal cell carcinoma as revealed by interactomics analysis. J Proteome Res.

[40] Yue D, Wang Y, Sun Y, Niu Y, Chang C (2017). C1QBP regulates YBX1 to suppress the androgen receptor (AR)-enhanced RCC cell invasion. Neoplasia..

[41] Wang Y, Fu D, Su J, Chen Y, Qi C, Sun Y (2017). C1QBP suppresses cell adhesion and metastasis of renal carcinoma cells. Sci Rep.

[42] Stratford AL, Habibi G, Astanehe A, Jiang H, Hu K, Park E (2007). Epidermal growth factor receptor (EGFR) is transcriptionally induced by the Y-box binding protein-1 (YB-1) and can be inhibited with Iressa in basal-like breast cancer, providing a potential target for therapy. Breast Cancer Res.

[43] Gao LJ, Gu PQ, Fan WM, Liu Z, Qiu F, Peng YZ (2011). The role of gC1qR in regulating survival of human papillomavirus 16 oncogene-transfected cervical cancer cells. Int J Oncol.

[44] Suzuki T, Oshiumi H, Miyashita M, Aly HH, Matsumoto M, Seya T (2013). Cell type-specific subcellular localization of phospho-TBK1 in response to cytoplasmic viral DNA. PLoS One.

[45] Gotoh K, Morisaki T, Setoyama D, Sasaki K, Yagi M, Igami K (2018). Mitochondrial p32/C1qbp is a critical regulator of dendritic cell metabolism and maturation. Cell Rep.

[46] Wang B, Heath-Engel H, Zhang D, Nguyen N, Thomas DY, Hanrahan JW (2008). BAP31 interacts with Sec61 translocons and promotes retrotranslocation of CFTRDeltaF508 via the derlin-1 complex. Cell..

[47] Kim KM, Adachi T, Nielsen PJ, Terashima M, Lamers MC, Kohler G (1994). Two new proteins preferentially associated with membrane immunoglobulin D. EMBO J.

[48] Adachi T, Schamel WW, Kim KM, Watanabe T, Becker B, Nielsen PJ (1996). The specificity of association of the IgD molecule with the accessory proteins BAP31/BAP29 lies in the IgD transmembrane sequence. EMBO J.

[49] Ng FW, Nguyen M, Kwan T, Branton PE, Nicholson DW, Cromlish JA (1997). p28 Bap31, a Bcl-2/Bcl-XL- and procaspase-8-associated protein in the endoplasmic reticulum. J Cell Biol.

[50] Ladasky JJ, Boyle S, Seth M, Li H, Pentcheva T, Abe F (2006). Bap31 enhances the endoplasmic reticulum export and quality control of human class I MHC molecules. J Immunol.

[51] Zen K, Utech M, Liu Y, Soto I, Nusrat A, Parkos CA (2004). Association of BAP31 with CD11b/CD18. Potential role in intracellular trafficking of CD11b/CD18 in neutrophils. J Biol Chem.

[52] Niu K, Xu J, Cao Y, Hou Y, Shan M, Wang Y (2017). BAP31 is involved in T cell activation through TCR signal pathways. Sci Rep.

[53] Seo SR, Lee HM, Choi HS, Kim WT, Cho EW, Ryu CJ (2017). Enhanced expression of cell-surface B-cell receptor-associated protein 31 contributes to poor survival of non-small cell lung carcinoma cells. PLoS One.

[54] Chen J, Guo H, Jiang H, Namusamba M, Wang C, Lan T (2019). A BAP31 intrabody induces gastric cancer cell death by inhibiting p27(kip1) proteasome degradation. Int J Cancer.

[55] Regan JA, Laimins LA (2008). Bap31 is a novel target of the human papillomavirus E5 protein. J Virol.

[56] Wang D, Grecula JC, Gahbauer RA, Schuller DE, Jatana KR, Biancamano JD (2006). p16 gene alterations in locally advanced squamous cell carcinoma of the head and neck. Oncol Rep.

[57] Xing XB, Cai WB, Luo L, Liu LS, Shi HJ, Chen MH (2013). The prognostic value of p16 Hypermethylation in Cancer: a meta-analysis. PLoS One.

[58] Herman JG, Merlo A, Mao L, Lapidus RG, Issa JP, Davidson NE (1995). Inactivation of the CDKN2/p16/MTS1 gene is frequently associated with aberrant DNA methylation in all common human cancers. Cancer Res.

[59] Lim AM, Do H, Young RJ, Wong SQ, Angel C, Collins M (2014). Differential mechanisms of CDKN2A (p16) alteration in oral tongue squamous cell carcinomas and correlation with patient outcome. Int J Cancer.

[60] Soria JC, Morat L, Commo F, Dabit D, Perie S, Sabatier L (2001). Telomerase activation cooperates with inactivation of p16 in early head and neck tumorigenesis. Br J Cancer.

[61] Burke LS, Hyland PL, Pfeiffer RM, Prescott J, Wheeler W, Mirabello L (2013). Telomere length and the risk of cutaneous malignant melanoma in melanoma-prone families with and without CDKN2A mutations. PLoS One.

[62] Padhi SS, Roy S, Kar M, Saha A, Roy S, Adhya A (2017). Role of CDKN2A/p16 expression in the prognostication of oral squamous cell carcinoma. Oral Oncol.

[63] Virmani AK, Muller C, Rathi A, Zoechbauer-Mueller S, Mathis M, Gazdar AF (2001). Aberrant methylation during cervical carcinogenesis. Clin Cancer Res.

[64] Dong SM, Kim HS, Rha SH, Sidransky D (2001). Promoter hypermethylation of multiple genes in carcinoma of the uterine cervix. Clin Cancer Res.

[65] Nuovo GJ, Plaia TW, Belinsky SA, Baylin SB, Herman JG (1999). In situ detection of the hypermethylation-induced inactivation of the p16 gene as an early event in oncogenesis. Proc Natl Acad Sci U S A.

[66] Wijetunga NA, Belbin TJ, Burk RD, Whitney K, Abadi M, Greally JM (2016). Novel epigenetic changes in CDKN2A are associated with progression of cervical intraepithelial neoplasia. Gynecol Oncol.

[67] Lee H, Lee EJ (2016). HPV infection and p16 promoter methylation as predictors of ASC-US/LSIL progression. Cancer Cytopathol.

[68] Vasiljevic N, Scibior-Bentkowska D, Brentnall AR, Cuzick J, Lorincz AT (2014). Credentialing of DNA methylation assays for human genes as diagnostic biomarkers of cervical intraepithelial neoplasia in high-risk HPV positive women. Gynecol Oncol.

[69] Tsitsiloni OE, Stiakakis J, Koutselinis A, Gogas J, Markopoulos C, Yialouris P (1993). Expression of alpha-thymosins in human tissues in normal and abnormal growth. Proc Natl Acad Sci U S A.

[70] Letsas KP, Frangou-Lazaridis M (2006). Surfing on prothymosin alpha proliferation and anti-apoptotic properties. Neoplasma..

[71] Jiang X, Kim HE, Shu H, Zhao Y, Zhang H, Kofron J (2003). Distinctive roles of PHAP proteins and prothymosin-alpha in a death regulatory pathway. Science..

[72] Vareli K, Frangou-Lazaridis M, van der Kraan I, Tsolas O, van Driel R (2000). Nuclear distribution of prothymosin alpha and parathymosin: evidence that prothymosin alpha is associated with RNA synthesis processing and parathymosin with early DNA replication. Exp Cell Res.

