# Supplementary figures and images for: Host proteome linked to HPV E7-mediated specific gene hypermethylation in cancer pathways
Source: Infect Agent Cancer. 2020 Feb 3;15:7. doi: 10.1186/s13027-020-0271-4 (PMC6998090; doi:10.1186/s13027-020-0271-4)

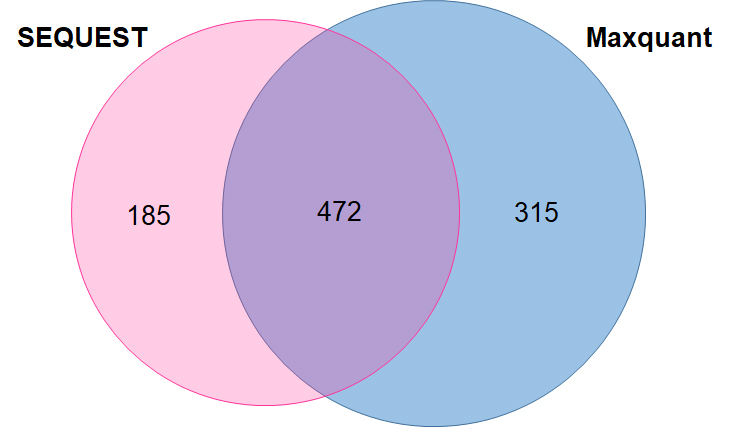

Supplement: Supplementary file 1 — Additional file 1 : Figure S1. Venn diagram of comparison of number of identified proteins with SEQUEST (Proteome Discoverer 2.2) and Maxquant database searching algorithm. The Venn diagram showed that Maxquant identified more proteins compared to SEQUEST but are mostly common. The commonly identified proteins by two algorithms were selected to further transcript abundance determination. [file 13027_2020_271_MOESM1_ESM.jpg]
